# Epidemiology of Sanfilippo syndrome: results of a systematic literature review

**DOI:** 10.1186/s13023-018-0796-4

**Published:** 2018-04-10

**Authors:** Tamás Zelei, Kata Csetneki, Zoltán Vokó, Csaba Siffel

**Affiliations:** 1 Syreon Research Institute, Budapest, Hungary; 2 Shire, 300 Shire Way, Lexington, MA 02421 USA

**Keywords:** Epidemiology, MPS III, Mucopolysaccharidosis type III, Sanfilippo syndrome, Systematic literature review

## Abstract

**Background:**

Sanfilippo syndrome (mucopolysaccharidosis [MPS] III subtypes A, B, C, and D) is a rare autosomal recessive inherited metabolic disorder that causes progressive neurocognitive degeneration. This systematic literature review was undertaken to compile and assess published epidemiological data, including various frequency measures and geographical variation on Sanfilippo syndrome.

**Methods:**

The following databases were systematically searched for terms related to Sanfilippo syndrome epidemiology: Medline, Embase, Cochrane Database of Systematic Reviews, Academic Search Complete, Cumulative Index to Nursing and Allied Health Literature, and the Centre for Reviews and Dissemination. Qualitative synthesis of research findings was performed.

**Results:**

Of 2794 publications found in the initial search, 116 were deemed eligible after title and abstract screening. Following full-text review, 46 papers were included in the qualitative synthesis. Results of this systematic literature review indicate that lifetime risk at birth ranges from 0.17–2.35 per 100,000 live births for all 4 subtypes of MPS III together, and from 0.00–1.62 per 100,000 live births for the most frequent subtype, MPS IIIA.

**Conclusion:**

All 4 subtypes of MPS III are exceptionally rare, but they each have devastating effects on children. Higher-quality epidemiological data are needed to appropriately target resources for disease research and management.

**Electronic supplementary material:**

The online version of this article (10.1186/s13023-018-0796-4) contains supplementary material, which is available to authorized users.

## Background

Sanfilippo syndrome (mucopolysaccharidosis [MPS] III) is a rare autosomal recessive inherited metabolic disorder that causes progressive neurocognitive degeneration. It consists of 4 subtypes (MPS IIIA, B, C, and D), each characterized by the deficiency of different enzymes that catalyze the metabolism of the glycosaminoglycan (GAG) heparan sulfate at the lysosomal level [1]. As a consequence of these deficiencies, GAG accumulates in the cells, resulting in progressive cellular damage affecting multiple organ systems and eventually leading to organ failure and cognitive decline [1]. Of the 4 subtypes, MPS IIIA (or Sanfilippo syndrome type A) is associated with the most severe symptoms and worst prognosis [2].

The disease initially presents itself with an onset of developmental or speech delay after a period of normal development, followed by severe behavioral problems and hyperactivity. Some children with MPS III present initial facial dysmorphic features, and parents may at first notice lags in language development or poor coordination in comparison with children of like age. With progressive cognitive decline, the patients eventually regress to a fully bedridden and vegetative state that results in significantly diminished life expectancy [3].

The number of new instances of Sanfilippo syndrome (all subtypes) is estimated at 1 in 70,000 live births [4], and overall point prevalence estimates range from 1 to 9 in 1,000,000 people [2]. The prevalence varies with geographic area, and certain subtypes appear to be predominant in specific regions of the world [2]. Overall, MPS IIIA and B are more commonly diagnosed than types C and D [2, 4].

Research into the epidemiology of Sanfilippo syndrome, as with other rare diseases, presents substantial challenges [5]. These include lack of central registration or referral systems, inapplicability of population sampling, large effects of random errors on occurrence probability, and inconsistent use of epidemiological terms. This systematic literature review was, therefore, undertaken to compile and assess published epidemiological data, including various frequency measures (e.g., prevalence, incidence, and lifetime risk) and geographical variation on Sanfilippo syndrome. In addition, we aimed to collect data on selected clinical characteristics and natural history of the disease available from papers included for review on the occurrence of Sanfilippo syndrome. For all parameters, we analyzed publications for all subtypes of MPS III and specifically for MPS IIIA. To our knowledge, this is the first systematic review of the scientific literature undertaken in this disease area.

## Methods

In performing this systematic review of the epidemiology of Sanfilippo syndrome, we used the following databases: Medline, Embase, Cochrane Database of Systematic Reviews, Academic Search Complete, Cumulative Index to Nursing and Allied Health Literature, and the Centre for Reviews and Dissemination. For each database search, we applied specific terms related to Sanfilippo syndrome epidemiology. We used the Sanfilippo syndrome–related terms (i.e., sanfilippo OR mpsiii OR mps3 OR ‘MPS III’ OR ‘MPS 3’ OR ‘MPS type III’ OR ‘MPS type 3’ OR mucopolysaccharidos* OR ‘sulfamidase deficiency’ OR ‘lysosomal storage disorder’) and combined them with epidemiological terms (i.e., inciden* OR prevalen* OR demograph* OR epidemiolog* OR frequen* OR rate OR distribut*; Additional file 1). The literature searches were performed in April and May 2016, and no language restriction or publication date limit was applied. In addition, for further qualifying studies we searched web pages of rare disease organizations, namely Orphanet, National Organization for Rare Disorders, Canadian Organization for Rare Disorders, and European Organisation for Rare Diseases. Snowball method was used to identify further studies in the reference lists of the included studies.

**Fig. 1:**
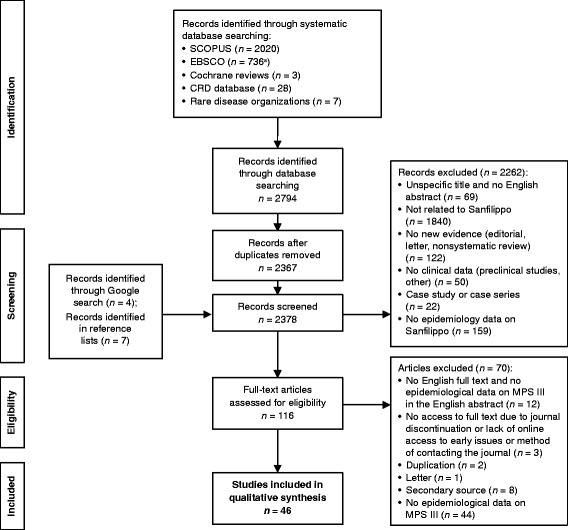
Flow diagram of included studies. ^a^Actual hit number was 744, but EBSCO automatically removed duplicates. *CRD* Centre for Reviews and Dissemination, *MPS* mucopolysaccharidosis

Titles and abstracts were screened for duplicates, and the papers were excluded based on predefined exclusion criteria (Fig. 1). The potentially relevant papers were reviewed in full text, and data were extracted for the following metrics of interest: the number of patients with Sanfilippo syndrome, ethnic background, patient’s age, patient’s age at diagnosis, survival, and clinical characteristics. We paid special attention to the calculation methods of epidemiological measures and categorized these according to the generally accepted definitions of the scientific community, disregarding the exact terms used in the papers (Table 1). We extracted dates pertaining to epidemiological measures (e.g., study period, date of point prevalence) and investigated the potential confounders, such as diagnostic methods, ethnicity founder effect, and inclusion of prenatal diagnoses.

**Table 1:** Definitions of epidemiological measures

Measure	Definition
Incidence rate	Number of new cases of a disease in a population during a given period of time divided by the total of the lengths of time that each individual in the population was at risk, expressed as person-time (e.g., person-years)
Cumulative incidence	Number of new cases of a disease in a population during a given period of time divided by the total number of people at risk of developing the disease at the beginning of the same period of time
Lifetime risk	Lifetime risk is a special case of cumulative incidence in which the period of time studied is the entire remaining lifetime; if one calculates the entire remaining lifetime from birth, the measure is called lifetime risk at birth
Point prevalence	Proportion of people in a population who have a disease or condition at a particular time point
Birth prevalence	Number of cases (including birth defects among live births, spontaneous fetal death, and induced terminations when available) divided by the total number of live births (or live births plus stillbirths)

The systematic literature review was conducted in compliance with the Preferred Reporting Items for Systematic Reviews and Meta-Analyses (PRISMA) statement, a generally accepted guideline for reporting systematic literature reviews [6]. The included papers were assessed for quality by the Strengthening the Reporting of Observational Studies in Epidemiology (STROBE) checklist, a guideline for reporting observational studies [7]. Papers of good, medium, poor, and very poor quality were defined as those that fulfilled > 80%, 66–80%, 50–65%, and < 50% of criteria, respectively.

## Results

Of 2794 publications found in the initial search, 116 were deemed eligible after title and abstract screening. Following full-text review, 46 papers were included in the qualitative synthesis (Fig. 1). These 46 papers reported data from 32 different countries. Most of the studies (93.5% [43/46]) were retrospective in design; 2 review papers and 1 retrospective study with prospective follow-up of identified patients were included in the current systematic review. The majority of the reported studies compared the number of identified patients with MPS III with the general population, defined as the number of live births in a given area during a specific study period. In 4 studies, the reference population with which the number of patients with MPS III was compared was defined as individuals with clinical suspicion of an inborn error of metabolism (IEM). The reference populations were based on clinical suspicion of a lysosomal storage disease (LSD) in 4 other studies, clinical suspicion of MPS in 1 study, and diagnosis of LSD in another. In these studies, only relative frequencies of MPS III (all types and subtypes) within the reference population were available. Four of the papers included only patients with MPS III in the analysis and the frequency of different subtypes was published.

The gold standard of MPS III diagnosis is an enzyme assay in cultured skin fibroblasts, leukocytes, plasma, or serum [8]. It was employed in 36 of the included studies. Mutational analyses were used in 1 study, and urinary GAG analysis was used in 4 studies. Five studies did not publish the method of MPS III diagnosis.

Quality assessment of the papers revealed that the reporting quality of epidemiology-related findings in these publications was generally low and highly heterogenic. Of the 46 papers included, 42 could be evaluated using the STROBE checklist. Seven of those were judged to be of good quality, 14 of medium quality, 14 of poor quality, and 7 of very poor quality (Additional file 2). Two review articles and 2 Spanish-language papers (English abstracts were available only) could not be evaluated using the STROBE checklist.

A variety of terms were used across the studies to report the proportion of newborns who were or would be affected by Sanfilippo syndrome. The majority of studies counted the number of diagnoses during a certain time period, including instances in which the diagnosis occurred later after birth. For these types of diseases, the proportion of newborns who are or will be affected can be best described as lifetime risk at birth [9]. To summarize, the included studies used 3 calculation methods to estimate lifetime risk at birth of Sanfilippo syndrome. The 2 most frequent calculation methods encountered were described previously [9]. The diagnosis (Dx) period method divides the number of patients with a particular (or specific) diagnosis in the observational period by the number of live births during the same period. The date-of-birth (DoB) method divides the number of individuals diagnosed with the condition by the total number of births during the period between the birth dates of the oldest and youngest patients (birth period) [9]. Three studies followed a cohort of newborns and counted the number of diagnosed patients within the same cohort [10–12]. This method can be considered as real lifetime risk at birth calculation if the follow-up period is long enough to diagnose all patients. Therefore, we use the terminology described above, disregarding the exact terms used in the papers.

### Epidemiology data for MPS III (all subtypes)

**Fig. 2:**
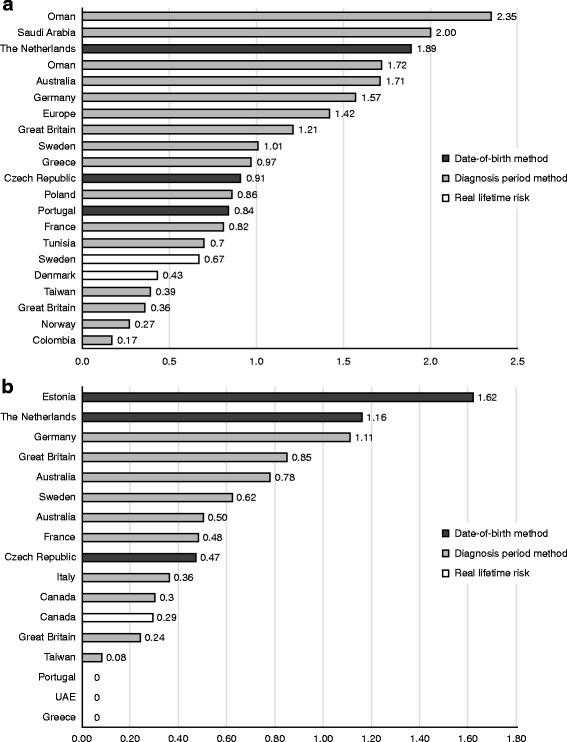
**a** Lifetime risk at birth of Sanfilippo syndrome, all subtypes by country/study (number of patients per 100,000 live births). **b** Lifetime risk at birth of Sanfilippo syndrome type A by country/study. *UAE* United Arab Emirates

Approximations for the lifetime risk at birth of MPS III (all subtypes) were reported in 17 publications for 18 countries or regions (Fig. 2a). Fourteen publications used the Dx method, while 3 publications used the DoB method (Table 2) [12–28]. Only 1 publication presented point prevalence data. In studies that compared lifetime risk at birth of the 4 subtypes of MPS III, type A was the most common, followed by type B. Type C was very rare, and few patients with type D were identified by the included studies (Table 3) [17, 19, 20, 23, 25–31].

**Table 2:** Reported lifetime risk at birth estimates of Sanfilippo syndrome (all subtypes)

First author, year	Country	Reporting quality of the paper	Study period	Representative of whole country?	Enzyme or mutational diagnosis?	Prenatal diagnosis included?	Evidence of ethnicity founder effect?	Sanfilippo all subtypes (sum of specific types)		
								Number of patients	Patients per 100,000 live births	Estimation method
Al-Maawali, 2012 [15]	Oman	Poor	1998–2007	No	Yes	No	No	All MPS III: 7	1.72	Dx
Angelis, 2015 [16]	Europe	Review	No data	Not applicable	Not applicable	–	–	–	1.42 (range, 0.36–2.00)	Dx
Baehner, 2005 [17]	Germany	Good	1980–1995	Yes	Yes	No	Yes (Turkish)	All MPS III: 211	1.57	Dx
Ben Turkia, 2009 [14]	Tunisia	Poor	1988–2005	Yes	Yes	No	No	All MPS III: 24	0.70	Dx
Gómez, 2012 [18]	Colombia	–	1998–2007	No	No data	No data	No data	–	0.17	Dx
Héron, 2011 [19]	France	Medium	1990–2006	Yes	Yes	No	No	All MPS III: 128	0.82^a^	Dx
Héron, 2011 [19]	Greece	Medium	1990–2006	Yes	Yes	No	No	All MPS III: 20 (including 1 unclassified)	0.97	Dx
Héron, 2011 [19]	Great Britain	Medium	1990–2006	Yes	Yes	No	No	All MPS III: 126 (including 6 unclassified)	1.21	Dx
Hult, 2014 [20]	Sweden	Medium	1990–2009	Yes	Yes	No	No	All MPS III: 21	1.01	Dx
Joshi, 2002 [21]	Oman	Poor	1998–2000	No	Yes	No	No	All MPS III: 3	2.35	Dx
Jurecka, 2015 [22]	Poland	Good	1970–2010	Yes	Yes	No data	No	All MPS III: 186	0.86	Dx
Lin, 2009 [23]	Taiwan	Good	1984–2004	Yes	Yes	No	No	All MPS III: 25	0.39	Dx
Malm, 2008 [12]	Norway	Medium	1979–2007	Yes	Yes	No data	No	All MPS III: 4	0.27	Real lifetime risk^b^
Malm, 2008 [12]	Denmark	Medium	1975–2007	Yes	Yes	No data	No	All MPS III: 8	0.43	Real lifetime risk^b^
Malm, 2008 [12]	Sweden	Medium	1975–2007	Yes	Yes	No data	No	All MPS III: 20	0.67	Real lifetime risk^b^
Moammar, 2010 [24]	Saudi Arabia	Poor	1983–2008	Yes	Yes	No	No	All MPS III: 3	2.00	Dx
Nelson, 1997 [25]	Great Britain	Poor	1958–1985	No	Yes	No	No	All MPS III: 3	0.36	Dx
Nelson, 2003 [26]	Australia	Poor	1969–1996	Yes	Yes	Yes	No	All MPS III: 11	1.71	Dx
Pinto, 2004 [27]	Portugal	Poor	1982–2001	No (Northern Portugal)	Yes	Yes	No	All MPS III: 14	0.84^c^	DoB
Poorthuis, 1999 [28]	The Netherlands	Poor	1970–1996	Yes	Yes	Yes	No	All MPS III: 156 (diagnosed between 1970 and 1996)	1.89^c^	DoB
Poupetová, 2010 [13]	Czech Republic	Medium	1975–2008	Yes	Yes	Yes	No	All MPS III: 24 (diagnosed between 1975 and 2008)	0.91^c^	DoB

^a^The original paper reported 0.73, which is possibly a misprint, as the sum of subtypes is 0.82

^b^Diagnosed patients among defined cohorts

^c^MPS III all subtypes but refers to the subtypes on different time horizons

*DoB* date-of-birth method, *Dx* diagnosis period method, *MPS* mucopolysaccharidosis

**Table 3:** Reported lifetime risk at birth estimates in studies included > 1 subtype of Sanfilippo syndrome

First author, year	Country	Study period	Reference population	Size of the reference population	Patients per 100,000 live births					
					Sum of all MPS III	Type A	Type B	Type C	Type D	Estimation method
Baehner, 2005 [17]	Germany	1980–1995	General population (live births in the study period)	13,410,924	1.57	1.11	0.37	0.10	0	Dx
Héron, 2011 [19]	France	1990–2006	General population (live births per year)	No data	0.82^a^	0.48	0.15	0.15	0.04	Dx
Hult, 2014 [20]	Sweden	1990–2009	General population (live births in the study period)	2,080,791	1.01	0.62	0.05	0.34	0	Dx
Lin, 2009 [23]	Taiwan	1984–2004	General population (live births in the study period)	6,377,299	0.39	0.08	0.28	0.03	0	Dx
Meikle, 1999 [29]	Australia	1980–1996	General population (live births in the study period)	No data	1.37^b^	0.78	0.43	0.07	0.09	Dx
Nelson, 1997 [25]	Great Britain	1958–1985	General population (live births in the study period in Northern Ireland)	839,517	0.36	0.24	0.12	0	0	Dx
Nelson, 2003 [26]	Australia	1969–1996	General population (live births in the study period in Western Australia)	641,179	1.71	0.62	0.78	0.16	0	Dx
Al-Jasmi, 2013 [30]	United Arab Emirates	1995–2010	General population	No data	–	0	1.05	0.25	–	DoB
Krabbi, 2012 [31]	Estonia	1985–2006	General population (live births in the study period)	370,298	1.62^b^	1.62	0	0	0	DoB
Pinto, 2004 [27]	Portugal	1982–2001	General population	No data	0.84	0	0.72	0.12	–	DoB
Poorthuis, 1999 [28]	The Netherlands	1970–1996	General population (live births in the study period)	Depends on the subtype of Sanfilippo: A: 6,972,344 (1960–1993); B: 11,131,609 (1940–1991); C: 7,119,276 (1949–1980); D: 2,994,743 (1970–1985)	1.89	1.16	0.42	0.21	0.10	DoB

^a^Original paper reported 0.73, which is possibly a misprint, as sum of subtypes is 0.82

^b^Calculated from the reported numbers of all subtypes

*DoB* date-of-birth method, *Dx* diagnosis period method, *MPS* mucopolysaccharidosis

The lowest lifetime risk at birth estimate, 0.17 per 100,000 live births, was found in a study from Colombia [18], while the highest estimate was reported in an Oman-based study with 2.35 patients per 100,000 live births [21]. However, those papers were deemed to have poor methodological and reporting quality. According to Malm et al., lifetime risk of MPS III at birth was reported as 0.27 per 100,000 in Norway, 0.43 per 100,000 in Denmark, and 0.67 per 100,000 live births in Sweden [12]. This study also estimated the point prevalence through diagnostic laboratory data for the same 3 Scandinavian countries at 0.88 per 1,000,000 inhabitants for Norway, 0.92 per 1,000,000 inhabitants for Denmark, and 1.63 per 1,000,000 inhabitants for Sweden. The paper was judged to have good methodological quality. Therefore, these results may serve as reliable estimates for the true occurrence of the disease in this region.

### Epidemiology data on subtypes of Sanfilippo syndrome

A total of 15 studies assessed the lifetime risk at birth of MPS IIIA (Additional file 3) [11, 13, 17, 19, 20, 23, 25–33]. The estimates ranged from 0.00 per 100,000 live births in the United Arab Emirates, Greece, and northern Portugal to 1.62 per 100,000 live births in Estonia (Fig. 2b).

Twelve studies reported the relative frequency of MPS IIIA within larger disease populations [19, 34–44] (Additional file 4). The relative frequency of MPS IIIA among all patients with MPS III was assessed in 4 studies [19, 34–36] and ranged from 19% (Brazil) to 71% (United Kingdom). MPS IIIA was reported in 3.6–38.4% of all instances of MPS (4 studies) [37–40], 1.9–7.9% of all LSD cases (3 studies) [41, 42, 44], and 4.2% of all IEM cases (1 study) [43].

Fifteen papers estimated lifetime risk at birth of MPS IIIB, and 9 of them were considered to be of medium or good reporting quality [13, 17, 19, 20, 23, 25–32, 45, 46]. In those studies, the highest estimated lifetime risk was 1.05 per 100,000 live births in the United Arab Emirates [30]. This estimate calls attention to the possibility of a founder mutation in isolated communities with a high degree of consanguinity. In this instance, high disease incidence was noted in 2 Emirati tribes. The estimates for lifetime risk at birth of MPS IIIB (number of patients per 100,000 live births) also were relatively high in Greece (0.78) [19] and Germany (0.37) [17], very low in Cuba (0.08) [46] and Sweden (0.05) [20], and no patients were diagnosed in Estonia during the 21-year study period [31].

The relative frequency of MPS IIIB among all patients with MPS III was assessed in 4 studies [19, 34–36] and ranged between 14% (France) [19] and 45.2% (Brazil) [35]. MPS IIIB was reported in 4.5–23.5% of all instances of MPS (5 studies) [37–40, 42], 1.7–17.0% of all instances of LSD (2 studies) [41, 44], and 2.1% of all instances of IEM (1 study) [43].

Fourteen papers estimated the lifetime risk at birth of MPS IIIC. Nine papers were considered to be of medium or good reporting quality [10, 13, 17, 19, 20, 23, 30–32]. Lifetime risk was between 0.00 and 0.42 per 100,000 live births in 10 of the 11 countries represented in those studies. A study assessing ethnic groups within the West Midlands region of the United Kingdom found a marked difference in the Northwestern European (1.16 per 100,000 live births) and Pakistani (10.38 per 100,000 live births) populations resident there.

Five studies published the relative frequency of MPS IIIC within larger disease groups [19, 34, 37, 40, 41] (Additional file 4). The relative frequency of MPS IIIC among all patients with MPS III was 13% in France [19] and 14.5% in Turkey [34]. MPS IIIC was reported in 2.9% (Turkey) [37] and 3.1% (Germany) [40] of all instances of MPS, and in 1.2% of all instances of LSD (India) [41].

A total of 7 papers attempted to determine lifetime risk of MPS IIID, and 3 had medium or good reporting quality [13, 19, 23]. In each of those studies, estimated lifetime risk at birth was below 0.10 per 100,000 live births. Owing to the low prevalence of MPS IIID, no studies assessed the relative frequencies of this subtype within larger disease groups.

### Summary of demographic and clinical characteristics

#### Age at diagnosis

Seven studies reported mean or median ages at diagnosis for patients with MPS III. For MPS IIIA, mean age at diagnosis in France, the United Kingdom, and Germany ranged from 3.5–4.9 years [19, 40]. Median age in Spain, Sweden, the Netherlands, and Australia ranged from 3.5–7.0 years [20, 29, 36, 47]. For patients with MPS IIIB, mean age at diagnosis ranged from 3.5–4.9 years in France, the United Kingdom, Greece, and Germany [19, 40], and median ages ranged from 2.5–3.5 years in Spain, Sweden, Australia, and Cuba [20, 29, 36, 46]. For studies in MPS IIIC populations, mean ages at diagnosis were between 4.5 and 19 years in 3 studies [19, 40, 48], and median ages at diagnosis were 7.0 and 10.7 years in 2 other papers [20, 29]. For MPS IIID, mean ages at diagnosis were 8.2 and 8.3 years in France and the United Kingdom, respectively [19]. In Australia, the median age at diagnosis of MPS IIID was 3.1 years [29].

#### Clinical characteristics – MPS IIIA

Three papers provided data on disease progression and occurrence of clinical manifestations for MPS IIIA [19, 36, 47]. Héron et al. reported the main clinical manifestations at diagnosis for 15 patients with MPS IIIA as language delay (93%), coarse features (92%), abnormal behavior (75%), hepatomegaly (51%), autism spectrum disorder (29%), and epilepsy (17%) [19]. Delgadillo et al. reported similar symptoms for 34 patients with MPS IIIA; speech delay, coarse facial features, and hyperactivity were the 3 most frequently occurring, with hyperactivity occurring at a median age of 3.8 years, speech loss at 5.8 years, epilepsy at 7.0 years (range, 2.5–16.0 years), and loss of walking ability at 10.4 years [36]. Valstar et al. found that the first signs of developmental delay and/or behavioral problems typically appeared at a median age of 2.5 years. Epilepsy was diagnosed for 53 of 80 patients at a median age of 11.0 years [47].

#### Clinical characteristics – MPS IIIB

Three papers published clinical characteristics of patients with MPS IIIB [19, 36, 49]. In the study by Héron et al., the most frequently occurring characteristics in 15 patients with MPS IIIB were similar to those for MPS IIIA: coarse features (94%), language delay (88%), abnormal behavior (69%), hepatomegaly (56%), autistic spectrum disorder (19%), and epilepsy (13%) [19]. Similar to MPS IIIA, in the study by Delgadillo et al., speech delay, coarse facial features, and hyperactivity were reported as the 3 most frequently occurring for 11 patients with MPS IIIB, with median age at appearance of 3.0 years for hyperactivity, 5.0 years for speech loss, 12.5 years (range, 5.5–37.0 years) for epilepsy, and 11.0 years for loss of walking ability [36]. A third study, published by van de Kamp et al., reported progression data for 23 patients with MPS IIIB. These researchers noted that the first disease signs appeared before the age of 4 years in 27% of patients, and dementia appeared before the age of 6 years in 24% of patients [49].

#### Clinical characteristics – MPS IIIC

Clinical characteristics were reported for patients with MPS IIIC in 3 publications [19, 48, 49]. The most frequently occurring clinical characteristics in 17 patients with MPS IIIC were reported by Héron et al. as language delay (92%), coarse features (85%), abnormal behavior (77%), hepatomegaly (39%), autism spectrum disorder (8%), and epilepsy (8%) [19]. Ruijter et al. reported that the first clinical signs and symptoms for patients with MPS IIIC appeared at a mean age of 3.5 years [48]. They included delayed speech development (92%), delayed motor development (83%), behavioral problems (83%), deterioration of speech (75%), sleeping problems (50%), diarrhea (58%), and deterioration of walking (17%). Van de Kamp et al. reported that the first signs appeared before the age of 4 years in 23% of 23 patients with MPS IIIC, and dementia appeared before the age of 6 years in 33% of patients [49].

#### Clinical characteristics – MPS IIID

No studies were identified in this literature search that included data on clinical characteristics and progression for patients with MPS IIID.

#### Survival

Mean survival for children with MPS IIIA was reported to be within the second decade of life (15.4 and 13 years) in 2 separate studies [19, 49], and the median survival values were reported as 15 and 18 years in 2 additional studies [1, 36]. Those with MPS IIIB appear to live slightly longer relative to patients with MPS IIIA; their mean survival times were reported as 17.1 and 19 years in 2 different studies [19, 49]. For patients with MPS IIIC, 3 studies reported mean survival of 19, 27.5, and 34 years [19, 48, 49]. No published survival data were found for patients with MPS IIID.

## Discussion

Although rarely encountered, the 4 subtypes of MPS III are characterized by genetic enzyme deficiencies causing progressive cognitive impairment and diminished behavioral capacity, ultimately leading to death in the second (type A and B) or third (type C) decade of life. No drugs are approved for treatment of the cognitive effects of MPS III, but gene therapies and enzyme replacements are being investigated. Due to the rarity of MPS III, broadly targeted population-based epidemiological studies have not been performed. This systematic literature review was performed to assess the existing evidence for incidence, prevalence, and lifetime risk at birth of each of the 4 subtypes of MPS III and summarizes the epidemiological findings related to the disease.

This systematic literature review found 46 papers that reported epidemiology data on Sanfilippo syndrome. Despite this fairly large number, only a small portion of these papers were characterized with good methodological and reporting quality. Results of this systematic literature review indicate that lifetime risk at birth ranges from 0.17–2.35 per 100,000 live births for all 4 subtypes of MPS III together, and 0.00–1.62 per 100,000 live births for MPS IIIA. The relative frequency of the MPS III subtypes are in agreement with previous reports, that is, among all subtypes, types A and B are more frequent than types C and D. These findings are consistent with the previous estimates of international organizations (mpssociety.org and orpha.net), but they also reveal a high degree of heterogeneity in the disease frequency estimates of different studies. The heterogeneity of the reported estimates is partly explained by different calculation methods, but other confounders such as type of diagnostic method, inclusion of prenatal diagnosis, and ethnicity founder effect can influence the results substantially. Taking into account these confounders, we can still assume differences in disease frequency in different countries.

Study methodologies are often inadequately described, and terms such as incidence, prevalence, and birth prevalence are frequently used inaccurately in the published literature. We propose to use lifetime risk at birth as a special case of cumulative incidence for the general measure of disease occurrence in diseases similar to Sanfilippo syndrome. In this systematic review, 3 methods were found for the estimation of lifetime risk at birth. The DoB method, which was found in several published studies, is vulnerable to bias depending on the dates bracketing the birth period. The Dx method was most frequently found in our review, and we propose to use this calculation method because it provides a more accurate estimation. The third method, which we termed real lifetime risk, covers large cohorts of patients who are followed for a long time, ideally long enough to diagnose and capture all patients within the cohorts. The disadvantage of this approach in the case of Sanfilippo syndrome is that the length of the follow-up period is difficult to determine because we found that the age range at diagnosis was relatively wide. In addition, we must emphasize that all of these methods depend on the effective case reporting.

As expected, this systematic review compiled substantial evidence of greatly diminished life expectancy in patients with MPS III. The published data showed death occurring at mean ages in the second decade of life with MPS IIIA and B, and in the third decade of life with MPS IIIC. No mortality data were found for MPS IIID. It is noted, however, that structured and summarized natural history and disease progression data for patients with any of the MPS III subtypes were only available in a small number of the identified studies.

This systematic review demonstrates the paucity of available data on the epidemiology of MPS III, although it is the most common of the mucopolysaccharidoses. Among epidemiologists, estimating prevalence of rare diseases presents distinct challenges. Studies of such conditions are not based on population sampling because investigators will not find a statistically appropriate number of patients in a random sample from the population at risk. Moreover, random error can have a large impact on the occurrence probability of a certain rare disease. In addition, Orphanet notes that epidemiology data for rare diseases may be affected by reliance on hospital data in regions that have established prevalence [5].

A recent study [50] investigated the epidemiology of the different types of mucopolysaccharidoses in Japan and Switzerland compared with similar data from other countries. The term ‘birth prevalence’ that the authors used in their calculation method was the Dx method of lifetime risk calculation, according to the terminology used in this paper. For MPS III (all subtypes) the authors reported 0.26 cases per 100,000 live births for Japan and 0.38 per 100,000 live births for Switzerland. These results are within the lower estimates of lifetime risk presented in the current review. The study did not find any additional papers that had not been included in this systematic review, and they did not investigate the lifetime risk of different subtypes of Sanfilippo syndrome.

Faced with limited, broad-based data, systematic literature reviews are useful in compiling available data and using the data to generate evidence on the epidemiology of rare diseases. Systematic collection and critical appraisal of published data can lower the risk of bias of individual studies, and provide validation for studies that report comparable results based on similar methods. Moreover, comparison of results from different countries and geographical areas can be performed with due diligence.

### Limitations

The epidemiological data compiled in this systematic literature review are based on methodologically diverse estimates of the impact of MPS III. Although the included studies reported actual patient numbers, population-based determinations of incidence and prevalence cannot be considered definitive. This is attributable to the heterogeneity of the estimates. As described previously, the identified studies suffer from inappropriate terminology applied to epidemiological measures; similar to the study performed by Foss et al. [9], we showed that nearly all previous estimates can be correctly interpreted as lifetime risk at birth. Due to the previously introduced between-study differences (statistical methodology, terminology, and diagnostic methods) the comparison of lifetime risk estimates across studies is limited.

A limitation of the presented natural history data is that our literature searches focused mainly on the disease frequency measures and there is a high chance that we missed potential papers publishing data on the natural history of Sanfilippo syndrome. A further potential limitation of our analysis is that we excluded non-English articles and articles for which the full text could not be accessed from this analysis.

## Conclusions

All 4 subtypes of MPS III are exceptionally rare genetic diseases, but they each have devastating effects on children. As research into pharmacological treatments for these diseases continues, higher-quality epidemiological data are needed to appropriately target resources.

## Additional files

Additional file 1: **Table S1.** Search strategies used for each database and the date of search in each. (DOCX 15 kb)

Additional file 2: STROBE Checklist for observational studies. (XLSX 82 kb)

Additional file 3: **Table S2.** Reported lifetime risk at birth estimates of Sanfilippo syndrome subtype A. File format: .docx. (DOCX 42 kb)

Additional file 4: **Table S3.** Relative frequency of Sanfilippo Type A within larger disease groups. **Table S4.** Relative frequency of Sanfilippo Type B within larger disease groups. **Table S5.** Relative frequency of Sanfilippo Type C within larger disease groups. (DOCX 57 kb)

## Abbreviations

DoB: Date-of-birth method;
Dx: Diagnosis period method
GAG: Glycosaminoglycan
IEM: Inborn error of metabolism
LSD: Lysosomal storage disorder
MPS: Mucopolysaccharidosis
PRISMA: Preferred Reporting Items for Systematic Reviews and Meta-Analyses
STROBE: Strengthening the Reporting of Observational Studies in Epidemiology
