# Supplementary material for: Epidemiology of Sanfilippo syndrome: results of a systematic literature review
Source: Orphanet J Rare Dis. 2018 Apr 10;13:53. doi: 10.1186/s13023-018-0796-4 (PMC5891921; doi:10.1186/s13023-018-0796-4)
Supplement: Supplementary file 1 — Table S1. Search strategies used for each database and the date of search in each. (DOCX 15 kb) [file 13023_2018_796_MOESM1_ESM.docx]

**Table S1** Search strategies used for each database and the date of search in each

| Database  (source) | Search terms | Date of search |
| --- | --- | --- |
| SCOPUS  (Medline, Embase) | TITLE-ABS-KEY ( ( mpsiii OR mps3 OR ‘MPS III’ OR ‘MPS 3’ OR ‘MPS type III’ OR ‘MPS type 3’ OR mucopolysaccharidos* OR sanfilippo OR ‘sulfamidase deficiency’ OR ‘lysosomal storage disorder’ ) AND ( inciden* OR prevalen* OR demograph* OR epidemiolog* OR frequen* OR rate OR distribut* ) ) | April 27, 2016 |
| EBSCO  (Academic Search Complete; CINAHL) | ( mpsiii OR mps3 OR ‘MPS III’ OR ‘MPS 3’ OR ‘MPS type III’ OR ‘MPS type 3’ OR mucopolysaccharidos* OR sanfilippo OR ‘sulfamidase deficiency’ OR ‘lysosomal storage disorder’ ) AND ( inciden* OR prevalen* OR demograph* OR epidemiolog* OR frequen* OR rate OR distribut* ) | April 27, 2016 |
| Cochrane reviews | (mpsiii OR mps3 OR ‘MPS III’ OR ‘MPS 3’ OR ‘MPS type III’ OR ‘MPS type 3’ OR mucopolysaccharidos* OR sanfilippo OR ‘sulfamidase deficiency’ OR ‘lysosomal storage disorder’ in Title, Abstract, Keywords) AND  (inciden* OR prevalen* OR demograph* OR epidemiolog* OR frequen* OR rate OR distribut* in Title, Abstract, Keywords) | May 13, 2016 |
| CRD database | (MPS) OR (mucopolysaccharidos*) OR (sanfilippo)  lysosomal storage disorder | May 13, 2016 |
| Rare disease organizations (Orphanet, NORD, CORD, EURORDIS) | Search of all relevant disease terms (e.g., Sanfilippo, MPS III, mucopolysaccharidosis III) | May 13, 2016 |

*CINAHL* Cumulative Index to Nursing and Allied Health Literature, *CORD* Canadian Organization for Rare Disorders, *CRD* Centre for Reviews and Dissemination, *EURORDIS* European Organization for Rare Diseases, *MPS* mucopolysaccharidosis, *NORD* National Organization for Rare Disorders
