# Supplementary material for: Epidemiology of Sanfilippo syndrome: results of a systematic literature review
Source: Orphanet J Rare Dis. 2018 Apr 10;13:53. doi: 10.1186/s13023-018-0796-4 (PMC5891921; doi:10.1186/s13023-018-0796-4)
Supplement: Supplementary file 3 — Table S2. Reported lifetime risk at birth estimates of Sanfilippo syndrome subtype A. File format: .docx. (DOCX 42 kb) [file 13023_2018_796_MOESM3_ESM.docx]

**Table S2** Reported lifetime risk at birth estimates of Sanfilippo syndrome subtype A

| First author, year | Country | Reporting quality of the paper | Study period | Representative of whole country? | Enzyme or mutational diagnosis? | Prenatal diagnosis included? | Evidence of ethnicity founder effect? | Sanfilippo type A | | |
| --- | --- | --- | --- | --- | --- | --- | --- | --- | --- | --- |
|  |  |  |  |  |  |  |  | Number of patients | Patients per 100,000 live births | Estimation method |
| Al-Jasmi, 2013 [[1](#_ENREF_1)] | United Arab Emirates | Medium | 1995–2010 | Yes | No | No | Yes | 0 | 0 | DoB |
| Applegarth, 2000 [[2](#_ENREF_2)] | Canada | Medium | 1972–1996 | No (British Columbia) | Yes | Yes | No | 3 | 0.29 | Real lifetime risk^a^ |
| Baehner, 2005 [[3](#_ENREF_3)] | Germany | Good | 1980–1995 | Yes | Yes | No | No | 149 | 1.11 | Dx |
| Dionisi-Vici, 2002 [[4](#_ENREF_4)] | Italy | Medium | 1985–1997 | Yes | Yes | No | No | 26 | 0.36 | Dx |
| Héron, 2011 [[5](#_ENREF_5)] | Greece | Medium | 1990–2006 | Yes | Yes | No | No | 0 | 0 | Dx |
| Héron, 2011 [[5](#_ENREF_5)] | France | Medium | 1990–2006 | Yes | Yes | No | No | 87 | 0.48 | Dx |
| Héron, 2011 [[5](#_ENREF_5)] | Great Britain | Medium | 1990–2006 | Yes | Yes | No | No | 89 | 0.85^b^ | Dx |
| Hult, 2014 [[6](#_ENREF_6)] | Sweden | Medium | 1990–2009 | Yes | Yes | No | No | 13 | 0.62 | Dx |
| Krabbi, 2012 [[7](#_ENREF_7)] | Estonia | Medium | 1985–2006 | Yes | No | No | No | 6 | 1.62 | DoB |
| Lin, 2009 [[8](#_ENREF_8)] | Taiwan | Good | 1984–2004 | Yes | Yes | No | No | 5 | 0.08 | Dx |
| Lowry, 1990 [[9](#_ENREF_9)] | Canada | Very poor | 1952–1986 | No (British Columbia) | No data | No | No | 4 | 0.30 | Dx |
| Meikle, 1999 [[10](#_ENREF_10)] | Australia | Poor | 1980–1996 | Yes | Yes | No | No | 33 postnatal (4 prenatal) | 0.78 (postnatal);  0.87 (postnatal + prenatal) | Dx |
| Nelson, 1997 [[11](#_ENREF_11)] | Great Britain | Poor | 1958–1985 | No (Northern Ireland) | Yes | No | No | 2 | 0.24 | Dx |
| Nelson, 2003 [[12](#_ENREF_12)] | Australia | Poor | 1969–1996 | No (Western Australia) | Yes | Yes | No | 4 | 0.50 | Dx |
| Pinto, 2004 [[13](#_ENREF_13)] | Portugal | Poor | 1982–2001 | No (Northern Portugal) | Yes | Yes | No | 0 | 0 | DoB |
| Poorthuis, 1999 [[14](#_ENREF_14)] | The Netherlands | Poor | 1970–1996 | Yes | Yes | Yes | No | 81 | 1.16 | DoB |
| Poupetová, 2010 [[15](#_ENREF_15)] | Czech Republic | Medium | 1975–2008 | Yes | Yes | Yes | No | 17 | 0.47 | DoB |

^a^Diagnosed patients among defined cohorts

^b^MPS III all types but refers to the subtypes on different time horizons

*DoB* date-of-birth method, *Dx* diagnosis period method

**References**

1. Al-Jasmi FA, Tawfig N, Berniah A, Ali BR, Hertecant JL, Bastaki F, et al. Prevalence and novel mutations of lysosomal storage disorders in United Arab Emirates: LSD in UAE. JIMD Rep. 2013;10:1–9.

2. Applegarth DA, Toone JR, Lowry RB. Incidence of inborn errors of metabolism in British Columbia, 1969–1996. Pediatrics. 2000;105:e10.

3. Baehner F, Schmiedeskamp C, Krummenauer F, Miebach E, Bajbouj M, Whybra C, et al. Cumulative incidence rates of the mucopolysaccharidoses in Germany. J Inherit Metab Dis. 2005;28:1011–7.

4. Dionisi-Vici C, Rizzo C, Burlina AB, Caruso U, Sabetta G, Uziel G, et al. Inborn errors of metabolism in the Italian pediatric population: a national retrospective survey. J Pediatr. 2002;140:321–7.

5. Héron B, Mikaeloff Y, Froissart R, Caridade G, Maire I, Caillaud C, et al. Incidence and natural history of mucopolysaccharidosis type III in France and comparison with United Kingdom and Greece. Am J Med Genet A. 2011;155A:58–68.

6. Hult M, Darin N, von Döbeln U, Månsson JE. Epidemiology of lysosomal storage diseases in Sweden. Acta Paediatr. 2014;103:1258–63.

7. Krabbi K, Joost K, Zordania R, Talvik I, Rein R, Huijmans JG, et al. The live-birth prevalence of mucopolysaccharidoses in Estonia. Genet Test Mol Biomarkers. 2012;16:846–9.

8. Lin H-Y, Lin S-P, Chuang C-K, Niu D-M, Chen M-R, Tsai F-J, et al. Incidence of the mucopolysaccharidoses in Taiwan, 1984–2004. Am J Med Genet A. 2009;149A:960–4.

9. Lowry RB, Applegarth DA, Toone JR, MacDonald E, Thunem NY. An update on the frequency of mucopolysaccharide syndromes in British Columbia. Hum Genet. 1990;85:389–90.

10. Meikle PJ, Hopwood JJ, Clague AE, Carey WF. Prevalence of lysosomal storage disorders. JAMA. 1999;281:249–54.

11. Nelson J. Incidence of the mucopolysaccharidoses in Northern Ireland. Hum Genet. 1997;101:355–8.

12. Nelson J, Crowhurst J, Carey B, Greed L. Incidence of the mucopolysaccharidoses in Western Australia. Am J Med Genet A. 2003;123A:310–3.

13. Pinto R, Caseiro C, Lemos M, Lopes L, Fontes A, Ribeiro H, et al. Prevalence of lysosomal storage diseases in Portugal. Eur J Hum Genet. 2004;12:87–92.

14. Poorthuis BJ, Wevers RA, Kleijer WJ, Groener JE, de Jong JG, van Weely S, et al. The frequency of lysosomal storage diseases in The Netherlands. Hum Genet. 1999;105:151–6.

15. Poupetová H, Ledvinová J, Berná L, Dvoráková L, Kozich V, Elleder M. The birth prevalence of lysosomal storage disorders in the Czech Republic: comparison with data in different populations. J Inherit Metab Dis. 2010;33:387–96.
