# Supplementary material for: Epidemiology of Sanfilippo syndrome: results of a systematic literature review
Source: Orphanet J Rare Dis. 2018 Apr 10;13:53. doi: 10.1186/s13023-018-0796-4 (PMC5891921; doi:10.1186/s13023-018-0796-4)
Supplement: Supplementary file 4 — Table S3. Relative frequency of Sanfilippo Type A within larger disease groups. Table S4. Relative frequency of Sanfilippo Type B within larger disease groups. Table S5. Relative frequency of Sanfilippo Type C within larger disease groups. (DOCX 57 kb) [file 13023_2018_796_MOESM4_ESM.docx]

**Table S3** Relative frequency of Sanfilippo Type A within larger disease groups

| First author, year | Country | Study design | Study period | Representative of whole country? | Enzyme or mutational diagnosis? | Reference population | Size of reference population | Sanfilippo type A | |
| --- | --- | --- | --- | --- | --- | --- | --- | --- | --- |
|  |  |  |  |  |  |  |  | Number of patients | Relative frequency |
| Church, 2013 [[1](#_ENREF_1)] | Turkey | Retrospective | 2009–2011 | No | Yes | Patients with a clinical suspicion of a LSD | 530 | 34 | 44.7% of all MPS III |
| Coelho, 1997 [[2](#_ENREF_2)] | Brazil | Retrospective | 1982  –1995 | No | Yes | Patients with a clinical suspicion of an IEM | 9,901 | 8 | 19% of all MPS III |
| Delgadillo, 2013 [[3](#_ENREF_3)] | Spain | Retrospective | 1971  –2010 | Yes | Yes | Patients with MPS III | 55 | 34 | 62% of all MPS III |
| Héron, 2011 [[4](#_ENREF_4)] | France | Retrospective, Prospective | 1990  –2006 | Yes | Yes | General population (number of live births per year) | NA | 87 | 68% of all MPS III |
| Héron, 2011 [[4](#_ENREF_4)] | Great Britain | Retrospective, Prospective | 1990–2006 | Yes | Yes | General population (number of live births per year) | NA | 89 | 71% of all MPS III |
| Héron, 2011 [[4](#_ENREF_4)] | Greece | Retrospective, Prospective | 1990–2006 | Yes | Yes | General population (number of live births per year) | NA | 0 | 0% of all MPS III |
| Emre, 2002 [[5](#_ENREF_5)] | Turkey | Retrospective | 1982  –2002 | No | Yes | Patients with a clinical suspicion of MPS | 42 | 5 | 14.71% of all MPS |
| Nelson, 1990 [[6](#_ENREF_6)] | Great Britain (Northern Ireland) | Retrospective | 1958–1985 | No | Yes | Cardiovascular patients with MPS | 22 | 3 | 13.6% of all MPS |
| Ozand, 1992 [[7](#_ENREF_7)] | Saudi Arabia | Retrospective | No data | Yes | Yes | Patients with a clinical suspicion of an IEM (children <12 years) | 910 | 2 (of 55 MPS patients) | 3.6% of all MPS |
| Sewell, 1988 [[8](#_ENREF_8)] | Germany | Retrospective | 1977–1987 | No | Yes | Undefined (Urine samples) | 5,255 | 61 | 38.4% of all MPS |
| Kadali, 2014 [[9](#_ENREF_9)] | India | Retrospective | 2007–2012 | Yes | Yes | Patients with a clinical suspicion of a LSD | 1,558 | 9 | 1.9% of all LSD |
| Krasnopolskaya, 1997 [[10](#_ENREF_10)] | Former Soviet Union | Retrospective | No data | Yes | Yes | Patients with a LSD | 445 | 35 | 7.9% of all LSD, 12.1% of all MPS |
| Michelakakis, 1995 [[11](#_ENREF_11)] | Greece | Retrospective | 1982–1995 | No | Yes | Patients with a clinical suspicion of a LSD | 2,745 | 3 | 3.2% of all LSD |
| Kagalwala, 1988 [[12](#_ENREF_12)] | India | Retrospective | No data | No | No | Patients with a clinical suspicion of an IEM | 4,604 | 2 | 4.2% of all IEM |

*IEM* inborn error of metabolism, *LSD* lysosomal storage disease, *MPS*, mucopolysaccharidosis, *MPS III*, mucopolysaccharidosis type III (Sanfilippo Syndrome), NA, not available

**Table S4** Relative frequency of Sanfilippo Type B within larger disease groups

| First author, year | Country | Study design | Study period | Representative of whole country? | Enzyme or mutational diagnosis? | Reference population | Size of reference population | Sanfilippo type B | |
| --- | --- | --- | --- | --- | --- | --- | --- | --- | --- |
|  |  |  |  |  |  |  |  | Number of patients | Relative frequency |
| Church, 2013 [[1](#_ENREF_1)] | Turkey | Retrospective | 2009–2011 | No | Yes | Patients with a clinical suspicion of a LSD | 530 | 31 | 40.8% of all MPS III |
| Coelho, 1997 [[2](#_ENREF_2)] | Brazil | Retrospective | 1982  –1995 | No | Yes | Patients with a clinical suspicion of an IEM | 9,901 | 19 | 45.2% of all MPS III |
| Delgadillo, 2013 [[3](#_ENREF_3)] | Spain | Retrospective | 1971  –2010 | Yes | Yes | Patients with MPS III | 55 | 11 | 20% of all MPS III |
| Héron, 2011 [[4](#_ENREF_4)] | France | Retrospective, Prospective | 1990  –2006 | Yes | Yes | General population (number of live births per year) | NA | 18 | 14% of all MPS III |
| Emre, 2002 [[5](#_ENREF_5)] | Turkey | Retrospective | 1982  –2002 | No | Yes | Patients with a clinical suspicion of MPS | 42 | 8 | 23.5% of all MPS |
| Krasnopolskaya, 1997 [[10](#_ENREF_10)] | Former Soviet Union | Retrospective | No data | Yes | Yes | Patients with a LSD | 445 | 13 (out of 290 MPS) | 4.5% of all MPS |
| Nelson, 1990 [[6](#_ENREF_6)] | Northern Ireland | Retrospective | 1958–1985 | Yes | Yes | Cardiovascular patients with MPS | 22 | 1 | 4.6% of all MPS |
| Ozand, 1992 [[7](#_ENREF_7)] | Saudi Arabia | Retrospective | No data | Yes | Yes | Patients with a clinical suspicion of an IEM (children <12 years) | 910 | 6 (of 55 MPS patients) | 10.9% of all MPS |
| Sewell, 1988 [[8](#_ENREF_8)] | Germany | Retrospective | 1977–1987 | No | Yes | Undefined (Urine samples) | 5,255 | 20 | 12.6% of all MPS |
| Kadali, 2014 [[9](#_ENREF_9)] | India | Retrospective | 2007–2012 | Yes | Yes | Patients with a clinical suspicion of a LSD | 1,558 | 8 | 1.7% of all LSD |
| Michelakakis, 1995 [[11](#_ENREF_11)] | Greece | Retrospective | 1982–1995 | No | Yes | Patients with a clinical suspicion of a LSD | 2,745 | 16 | 17% of all LSD |
| Kagalwala, 1988 [[12](#_ENREF_12)] | India | Retrospective | No data | No | No | Patients with a clinical suspicion of an IEM | 4,604 | 1 | 2.1% of all IEM |

*IEM* inborn error of metabolism, *LSD* lysosomal storage disease, *MPS*, mucopolysaccharidosis, *MPS III*, mucopolysaccharidosis type III (Sanfilippo Syndrome), NA, not available

**Table S5** Relative frequency of Sanfilippo Type C within larger disease groups

| First author, year | Country | Study design | Study period | Representative of whole country? | Enzyme or mutational diagnosis? | Reference population | Size of reference population | Sanfilippo type C | |
| --- | --- | --- | --- | --- | --- | --- | --- | --- | --- |
|  |  |  |  |  |  |  |  | Number of patients | Relative frequency |
| Church, 2013 [[1](#_ENREF_1)] | Turkey | Retrospective | 2009–2011 | No | Yes | Patients with a clinical suspicion of a LSD | 530 | 11 | 14.5% of all MPS III |
| Héron, 2011 [[4](#_ENREF_4)] | France | Retrospective, Prospective | 1990  –2006 | Yes | Yes | General population (number of live births per year) | NA | 17 | 13.3% of all MPS III |
| Emre, 2002 [[5](#_ENREF_5)] | Turkey | Retrospective | 1982  –2002 | No | Yes | Patients with a clinical suspicion of MPS | 42 | 1 | 2.9% of all MPS |
| Sewell, 1988 [[8](#_ENREF_8)] | Germany | Retrospective | 1977–1987 | No | Yes | Undefined (Urine samples) | 5,255 | 5 | 3.1% of all MPS |
| Kadali, 2014 [[9](#_ENREF_9)] | India | Retrospective | 2007–2012 | Yes | Yes | Patients with a clinical suspicion of a LSD | 1,558 | 6 | 1.2% of all LSD |

*IEM* inborn error of metabolism, *LSD* lysosomal storage disease, *MPS*, mucopolysaccharidosis, *MPS III*, mucopolysaccharidosis type III (Sanfilippo Syndrome), NA, not available

**References**

1. Church H, Petty J, Righart J, Parkes O, Egerton C, Savage W, et al. The incidence of mucopolysaccharidoses and related disorders in the Turkish population: a 3 year study. Mol Genet Metab. 2013;108:S30.

2. Coelho JC, Wajner M, Burin MG, Vargas CR, Giugliani R. Selective screening of 10,000 high-risk Brazilian patients for the detection of inborn errors of metabolism. Eur J Pediatr. 1997;156:650–4.

3. Delgadillo V, del Mar O'Callaghan M, Gort L, Coll MJ, Pineda M. Natural history of Sanfilippo syndrome in Spain. Orphanet J Rare Dis. 2013;8:189.

4. Héron B, Mikaeloff Y, Froissart R, Caridade G, Maire I, Caillaud C, et al. Incidence and natural history of mucopolysaccharidosis type III in France and comparison with United Kingdom and Greece. Am J Med Genet A. 2011;155A:58–68.

5. Emre S, Terzioğlu M, Coşkun T, Tokath A, Ozalp I, Müller V, et al. Biochemical and molecular analysis of mucopolysaccharidoses in Turkey. Turk J Pediatr. 2002;44:13–7.

6. Nelson J, Shields MD, Mulholland HC. Cardiovascular studies of the mucopolysaccharidoses. J Med Genet. 1990;27:94–100.

7. Ozand PT, Devol EB, Gascon GG. Neurometabolic diseases at a national referral center: five years experience at the King Faisal Specialist Hospital and Research Centre. J Child Neurol. 1992;(7)(Suppl):S4–11.

8. Sewell AC. Urinary screening for disorders of heteroglycan metabolism: results of 10 years experience with a comprehensive system. Klin Wochenschr. 1988;66:48–53.

9. Kadali S, Kolusu A, Gummadi MR, Undamatia J. The relative frequency of lysosomal storage disorders: a medical genetics referral laboratory's experience from India. J Child Neurol. 2014;29:1377–82.

10. Krasnopolskaya XD, Mirenburg TV, Akhunov VS, Voskoboeva EY. Postnatal and prenatal diagnosis of lysosomal storage diseases in the former Soviet Union. Wiener Klinische Wochenschrift. 1997;109:74–80.

11. Michelakakis H, Dimitriou E, Tsagaraki S, Giouroukos S, Schulpis K, Bartsocas CS. Lysosomal storage diseases in Greece. Genet Couns. 1995;6:43-7.

12. Kagalwala TY, Bharucha BA, Kumta NB, Naik GG. The mucopolysaccharidoses: a study of 48 cases. Indian J Pediatr. 1988;55:919–25.
